# From Chlamydia to Gonococcus: Emerging Trends of Infectious Conjunctivitis and Keratoconjunctivitis in a Tertiary Ophthalmology Center in Switzerland from 2005–2025

**DOI:** 10.3390/microorganisms14091989

**Published:** 2026-09-08

**Authors:** Sophia Näther, Jan Polzer, Chantal Quiblier, Hendrik Koliwer-Brandl, Frank Imkamp, Oliver Nolte, Daniel Barthelmes, Mario Damiano Toro, Mohammadjavad Abbasi, Sadiq Said, Frank Blaser

**Affiliations:** 1 Department of Ophthalmology, University Hospital Zurich, University of Zurich, 8091 Zurich, Switzerland; sophia.naether@usz.ch (S.N.);; 2 Institute of Medical Microbiology, University of Zurich, 8006 Zurich, Switzerlandonolte@imm.uzh.ch (O.N.); 3 The Save Sight Institute, The University of Sydney, Sydney, NSW 2000, Australia; 4 Eye Clinic, Public Health Department, Federico II University, 80138 Naples, Italy; mariodamiano.toro@unina.it; 5 School of Medicine and Surgery, University of Naples Federico II, 80138 Naples, Italy; mj.abbasi@live.com; 6 Augenklinik Wettingen, 5430 Wettingen, Switzerland

**Keywords:** gonococcal conjunctivitis, gonococcal keratoconjunctivitis, chlamydial conjunctivitis, sexually transmitted infections, corneal perforation

## Abstract

Gonococcal and chlamydial conjunctivitis and keratoconjunctivitis are potentially sight-threatening ocular manifestations of sexually transmitted infections. Recent epidemiological data indicate rising rates of gonorrhoea and chlamydia infections worldwide, raising concerns about a possible increase in associated ocular infections. This retrospective single-center study analyzed the frequency and temporal trends of laboratory-confirmed gonococcal and chlamydial ocular infections in adults treated at the University Hospital Zurich between January 2005 and July 2025. Clinical characteristics, treatment, complications, and outcomes were additionally assessed for gonococcal cases. We identified 17 gonococcal and 121 chlamydial conjunctivitis or keratoconjunctivitis cases over the past approximately 20.5 years. A marked increase in gonococcal cases was observed between 2021 and 2025 (*p* < 0.0001), during which 70.6% of all cases were diagnosed. Complications occurred in 41.1% of gonococcal infections, including one corneal perforation requiring keratoplasty. Although gonococcal conjunctivitis and keratoconjunctivitis remain rare, their recent increase and risk of severe ocular complications highlight the importance of early recognition and prompt treatment. In contrast, chlamydial conjunctivitis was more common, affected a younger population, and showed a significant declining trend, with an average annual decrease of 5% (*p* = 0.0015).

## 1. Introduction

Chlamydia and gonorrhoea are the most common bacterial sexually transmitted diseases (STDs) worldwide, with significant health implications [1,2].

Gonorrhoea can cause symptoms in the genitals, anus, or throat, and untreated infections can lead to severe complications in men and women [1]. Transmission occurs through direct mucosal contact, such as during sexual activity or childbirth [3,4,5]. In neonates, untreated gonococcal conjunctivitis can lead to severe complications, which may lead to corneal ulceration and blindness [3]. In adults, gonococcal conjunctivitis typically results from autoinoculation or sexual transmission when infected genital secretions come into direct contact with the ocular mucosa [4,6,7]. Clinically, gonococcal conjunctivitis presents with rapid-onset copious purulent discharge, frequently accompanied by eyelid swelling, conjunctival injection, and pain. If left untreated, the infection can spread to the cornea, leading to keratitis, anterior chamber inflammation, and permanent visual impairment [8]. The ability of gonococcus to penetrate the intact corneal epithelium may lead to corneal perforation [9,10]. Diagnostic measures beyond the clinical appearance include bacterial culture, direct fluorescence, and PCR, with PCR being the most sensitive and specific method [2,4]. Recent epidemiological data indicate a rising incidence of gonococcal keratoconjunctivitis in Europe [11,12]. Due to the alarming rise in antimicrobial resistance, the WHO classified *Neisseria gonorrhoeae* (*N. gonorrhoeae*) in 2017 as a high-priority pathogen for which new antibiotics are urgently needed [13]. In most countries, ceftriaxone remains the only effective option for an empirical monotherapy; however, sporadic cases of treatment failure and resistance to third-generation cephalosporins have been reported worldwide [14,15]. In the UK, a targeted, opportunistic vaccination programme using the 4CMenB (Bexsero) vaccine has been introduced for individuals at increased risk of gonorrhoea, although protection against gonorrhoea remains an off-label indication [16]. Table A1 summarizes the current international treatment recommendations for gonococcal conjunctivitis in adults.

*Chlamydia trachomatis* (*C. trachomatis*), on the other hand, is the most common bacterial STD worldwide [17,18]. Ocular *C. trachomatis* infections can be classified into three entities [19,20]. Trachoma, caused by serovars A–C, is the leading infectious cause of blindness in developing countries [21]. Neonatal conjunctivitis develops in 20–50% of infants born to mothers with chlamydial cervical infection [22]. Adult chlamydial conjunctivitis occurs in sexually active individuals, and up to 80% of affected patients have a concurrent genital infection with serovars D–K [23]. Rarely, adult inclusion conjunctivitis may be acquired through exposure to contaminated, incompletely chlorinated swimming pool water, giving rise to the historical term “swimming pool conjunctivitis.” However, contemporary evidence does not support waterborne transmission as a relevant route of infection. Instead, adult chlamydial conjunctivitis is now understood to be almost exclusively sexually acquired [20]. Although chlamydial keratoconjunctivitis is often less acutely symptomatic than gonococcal infections, it can still lead to significant ocular morbidity [24]. The patients usually present with mild to moderate symptoms, such as chronic conjunctival injection, mild discharge, and photophobia [25]. Chronic or recurrent cases can lead to corneal scarring, which may result in impaired vision and blindness if left untreated [25]. Diagnostic measures are similar to gonorrhoea, with PCR being the standard, offering high sensitivity and specificity [26]. Treatment typically involves oral antibiotics, with doxycycline and azithromycin being the most commonly recommended [17]. Antibiotic resistance in *C. trachomatis* remains relatively rare; however, emerging concerns regarding treatment failures due to non-compliance or incorrect dosing highlight the need for continued vigilance in treatment and management.

The World Health Organization (WHO) reports that in 2020 an estimated 128.5 million (90.0–173.8 million) new chlamydia infections and 82.4 million (47.7–130.4 million) new gonorrhoea infections occurred among adolescents and adults aged 15–49 years worldwide [27]. The European Centre for Disease Prevention and Control reported that the 2023 gonorrhoea notification rate in the EU/EEA (European Union/European Economic Area) was the highest observed since the initiation of European sexually transmitted infection surveillance in 2009. Between 2014 and 2023, this rate increased by 321% [28].

In Switzerland, chlamydial and gonococcal infections are subject to mandatory notification to the Federal Office of Public Health (FOPH). Laboratories report confirmed cases directly to the FOPH via the electronic notification platform. In the case of a positive gonococcal result, physicians are additionally required to submit a supplementary clinical notification form, which must be forwarded to the competent cantonal physician within one week. However, although the surveillance system provides comprehensive data on the incidence of laboratory-confirmed infections, neither laboratory nor physician reporting forms routinely capture information on ocular manifestations or site-specific clinical presentations. Consequently, the burden, clinical characteristics, and epidemiology of ocular *C. trachomatis* and *N. gonorrhoeae* infections remain largely unknown in Switzerland. Reported *C. trachomatis* infections in Switzerland increased markedly between 2000 and 2022, with a brief interruption during the first year of the COVID-19 pandemic, followed by a slight decline in 2023 and stabilization in 2024 (incidence of 142.1 per 100,000 inhabitants). In contrast, gonococcal infections have continued to rise, reaching an incidence of 75.6 per 100,000 inhabitants in 2024, with a particularly pronounced increase among men, especially MSM aged 25–34 years [29]. Despite these increasing notification rates, no national surveillance data are available on ocular involvement, highlighting an important knowledge gap.

This study analyzes the frequency and trends of gonococcal and chlamydial conjunctivitis and keratoconjunctivitis at a large Swiss university hospital over approximately 20.5 years (2005–2025). By evaluating all documented cases, we aim to provide valuable insights into the evolving landscape of these ocular infections.

## 2. Materials and Methods

This is an investigator-initiated, single center, retrospective study conducted at the Department of Ophthalmology at the University Hospital Zurich in Switzerland. The design, conduct and reporting of this study were guided by the STROBE (Strengthening the Reporting of Observational Studies in Epidemiology) guidelines for observational studies. We identified patients diagnosed with PCR- or culture-positive gonococcal or chlamydial ocular infections in our tertiary care center between January 2005 and July 2025. The leading ethics committee in Zurich waived our study protocol (BASEC number Req-2025-00917, Business Administration System for Ethics Committees, the Swiss Ethics–application platform) as it did not fall within the scope of the Human Research Act. Nevertheless, all data were handled according to Good Clinical Practice guidelines.

### 2.1. Data Collection

The data query for positive results in the conjunctival swabs for *N. gonorrhoeae* and *C. trachomatis* (PCR and/or culture; culture not available for *C. trachomatis*) of adults aged 16 and older was performed by the Institute of Medical Microbiology of the University of Zurich. Data were transmitted in anonymized form, including swab date, gender and age. Due to the low number of positive gonococcal cases and their clinical severity, these cases were already known to the Department of Ophthalmology at the time of reporting. We reviewed our electronic medical files of these patients regarding clinical manifestation, treatment and outcome. The Cantonal Ethics Committee approved this additional data analysis of the clinical characteristics of these cases upon request.

### 2.2. Pathogen Detection

For the detection of *C. trachomatis* and/or *N. gonorrhoeae*, clinical samples were routinely processed using one of two parallel laboratory workflows. Most samples were processed by a Cobas 4800 (Roche^®^, Basel, Switzerland) for DNA extraction and PCR setup, with PCR subsequently performed on a Cobas Z instrument. A second workflow, based on sample processing on a QIAsymphony SP/AS instrument (Qiagen^®^, Hilden, Germany) followed by bacterial broad-spectrum PCR on a Lightcycler 480-II (Roche^®^, Basel, Switzerland), was additionally established approximately 10 years ago and has since been used in parallel with the Cobas 4800 workflow [30]. QIAsymphony/LightCycler workflow did not replace the Cobas 4800 workflow during the study period. The two workflows have comparable analytical sensitivity for the detection of *C. trachomatis* and *N. gonorrhoeae*.

For cultural identification of *N. gonorrhoeae*, 100 µL of specimen was inoculated onto Chocolate PolyViteX agar (PVX; bioMérieux, Marcy-l’Étoile, France), a non-selective agar for fastidious bacteria, and Thayer Martin agar (VCA3; bioMérieux, Marcy-l’Étoile, France), a selective agar for *N. gonorrhoeae* containing antibiotics to inhibit normal flora. Plates were incubated up to 48 h at 37 °C with 7.5% CO_2_. Grown bacteria were further identified using the direct transfer–formic acid method and matrix-assisted laser desorption/ionization time-of-flight mass spectrometry (MALDI-TOF MS; Bruker, Bremen, Germany), enabling rapid and accurate species identification based on ribosomal protein mass spectra [31].

### 2.3. Statistical Analysis

We applied descriptive statistics, presenting medians with interquartile ranges (IQRs), or minimum to maximum values for continuous data, and numbers and percentages for categorical data.

Comparisons between groups were performed using the Mann–Whitney U test for continuous variables and Fisher’s exact test for categorical variables.

Temporal trends in annual case counts were analyzed using a hierarchical analytical strategy. Poisson regression was used as the primary test for trend estimation, providing incidence rate ratios (IRRs) and average annual percentage changes with 95% confidence intervals. To confirm the robustness of the observed pattern and to identify a potential change point, we applied the Mann–Kendall test (for monotonic trends) and the Pettitt change-point test as sensitivity analyses. A post hoc comparison of case counts between two clinically meaningful epochs (2005–2020 vs. 2021–2025) was performed using the Mann–Whitney U test. Overdispersion was checked for the Poisson models using the ratio of residual deviance to degrees of freedom and was not observed (dispersion parameter < 1.2). Given the small sample size for gonococcal cases, we acknowledge that these exploratory analyses may be subject to overinterpretation; results should be interpreted with caution. All statistical tests were two-sided, and a *p*-value < 0.05 was considered statistically significant.

Statistical analyses were performed using R version 4.4.2 (R for Statistical Computing, Vienna, Austria).

## 3. Results

### 3.1. Study Population and Descriptive Characteristics

Between January 2005 and July 2025, we identified a total of 17 patients with gonococcal conjunctivitis/keratoconjunctivitis and 121 patients with chlamydial conjunctivitis. The chlamydia group had a median age of 26 years (IQR 20–34, range 16–74) and a male predominance (83/121, 68.6%). The gonococcus group was older (median 44 years, IQR 30–58, range 21–79) and was predominantly male (15/17, 88.2%). Demographic characteristics are summarized in Table 1.

### 3.2. Gonococcal Conjunctivitis/Keratoconjunctivitis

**Figure 1:**
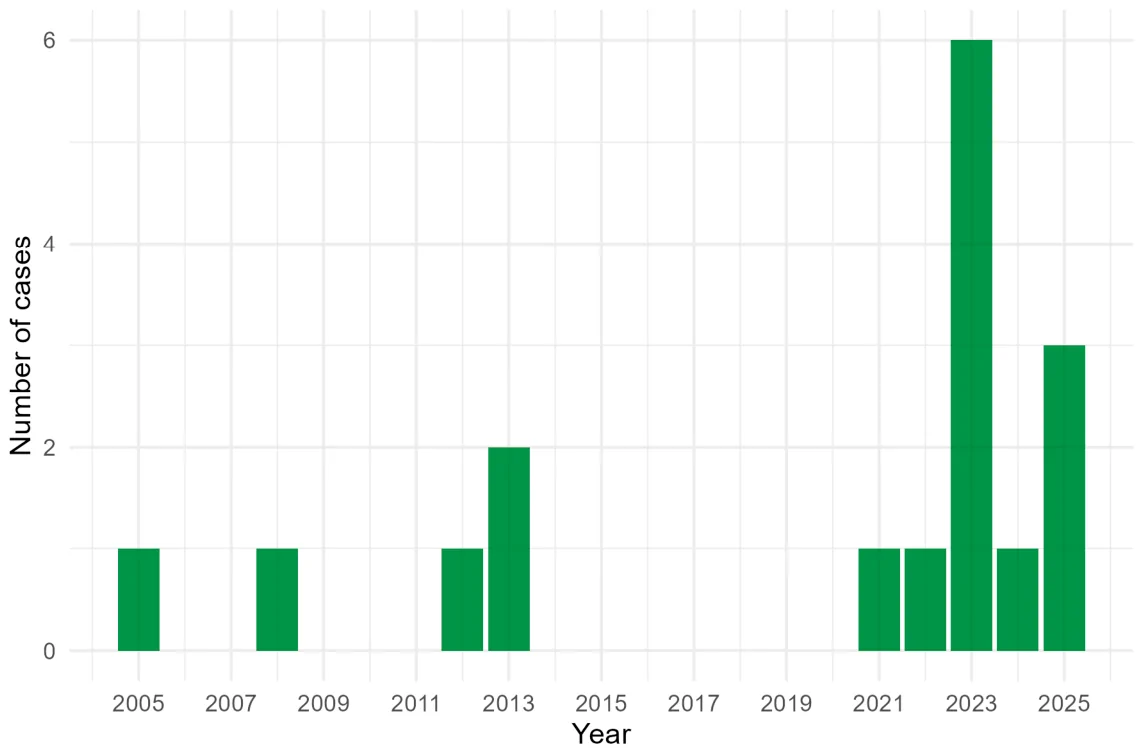
Annual number of gonococcal conjunctivitis and keratoconjunctivitis during the study period of 2005 to 2025.

Reviewing the medical records, we identified 20 eyes of 17 patients with PCR- or culture-confirmed gonococcal conjunctivitis or keratoconjunctivitis. Between 2005 and 2020, only five cases occurred over 16 years (mean annual incidence 0.31 cases/year). In contrast, between 2021 and 2025, 12 cases were recorded over five years (mean 2.4 cases/year) (Table 2). The exact Poisson test comparing the two periods yielded an incidence rate ratio (IRR) of 7.68 (95% CI: 2.52 to 27.83, *p* < 0.0001), indicating a highly significant increase in recent years. The highest annual incidence was recorded in 2023, when six cases (35.3%) were diagnosed. Figure 1 displays the annual case distribution of gonococcal infections.

**Figure 2:**
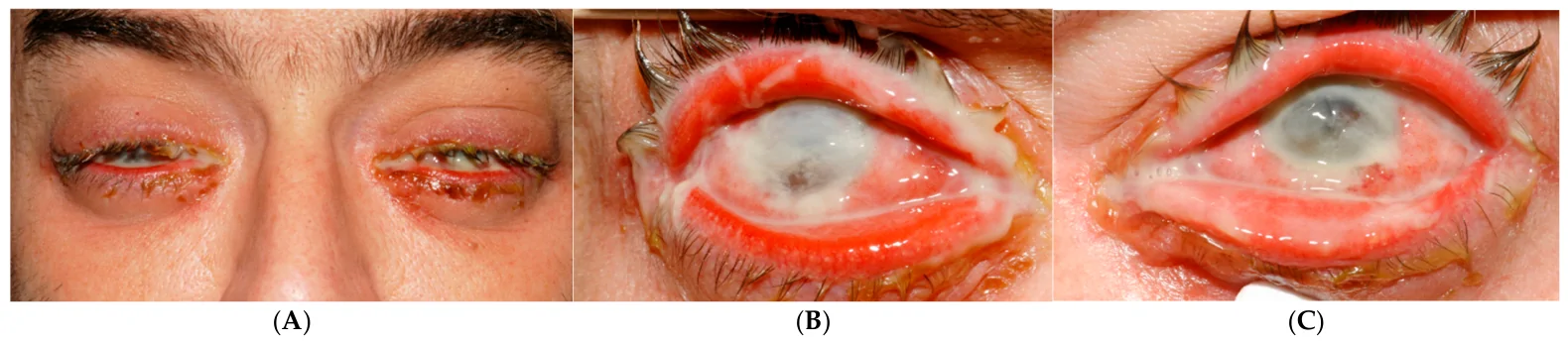
Example of one case with bilateral gonococcal keratoconjunctivitis and severe corneal involvement ((**A**)—overview, (**B**)—right eye, (**C**)—left eye).

**Figure 3:**
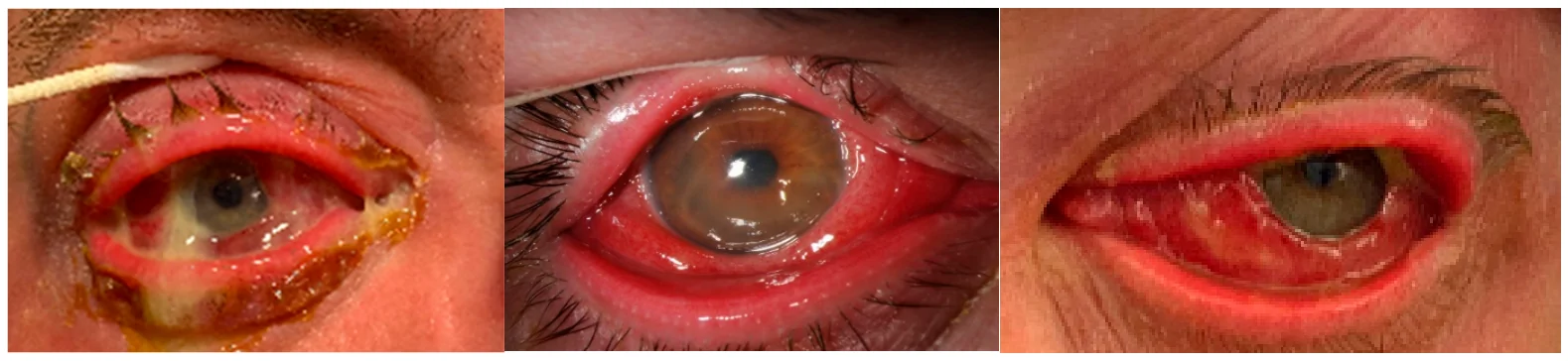
Examples of clinical presentation of three different cases at initial visit with eyelid swelling, conjunctival injection and chemosis, mucopurulent discharge.

Disease was unilateral in 14 (82.4%) patients; three patients presented with bilateral involvement (cases 1, 6, and 7; example Figure 2). All patients presented with marked eyelid edema, conjunctival hyperemia, and copious mucopurulent discharge (Figure 3).

Because of severe periorbital swelling and pain, six patients (35.3%) underwent computed tomography (CT), which demonstrated pre-septal soft-tissue swelling without post-septal involvement in all cases. We documented corneal involvement in 9 (52.9%) patients, including erosions (*n* = 2), infiltrates (*n* = 3), corneal edema (*n* = 2), corneal ulceration (*n* = 1), and corneal perforation (*n* = 1).

At initial presentation, clinicians suspected bacterial conjunctivitis in nine (52.9%) patients. A sexual history positive for recent unprotected intercourse with new or multiple partners was documented in seven (41.1%) patients, including MSM (men who have sex with men) status in four (23.5%). Furthermore, four (23.5%) patients reported concurrent urogenital symptoms. We confirmed urogenital co-infection in three (17.6%) patients, including one simultaneous *C. trachomatis* and *N. gonorrhoeae* infection. In one patient with gonococcal conjunctivitis, only *C. trachomatis* was detected in the urethral swab. In one patient with urogenital symptoms, the three-glass test was performed after completion of antibiotic therapy and was therefore negative (case 11). Conjunctival co-infection with *C. trachomatis* was detected in two patients (11.8%, cases 6 and 11).

Regarding the systemic antibiotic treatment, 16 of 17 (94.1%) patients received systemic ceftriaxone antibiotic treatment. In the patient who did not receive systemic ceftriaxone (case no. 2), an initial suspicion of post-septal cellulitis led to inpatient admission and intravenous treatment with co-amoxicillin. Due to a rapid clinical improvement, we discharged the patient two days later. The culture result of the conjunctival swab subsequently tested positive for gonococci; however, as the patient was asymptomatic, no further treatment with ceftriaxone was administered. Three (17.6%) patients were systemically treated for gonococcal infection on the day of presentation. The remaining cases began treatment within one to 12 days. Patients with severe disease received repeated intravenous ceftriaxone. To address suspected or confirmed *C. trachomatis* co-infection, four patients received azithromycin and four received doxycycline. Additional agents were used selectively, including co-amoxicillin, metronidazole, meropenem, and acyclovir. Topical therapy varied and included fluoroquinolones or fortified antibiotics. Four patients received topical corticosteroids, and two patients required adjunctive autologous serum eye drops. Hospital admission was required in 8 patients; one of these patients (case no. 10) declined hospitalization against medical advice.

**Figure 4:**
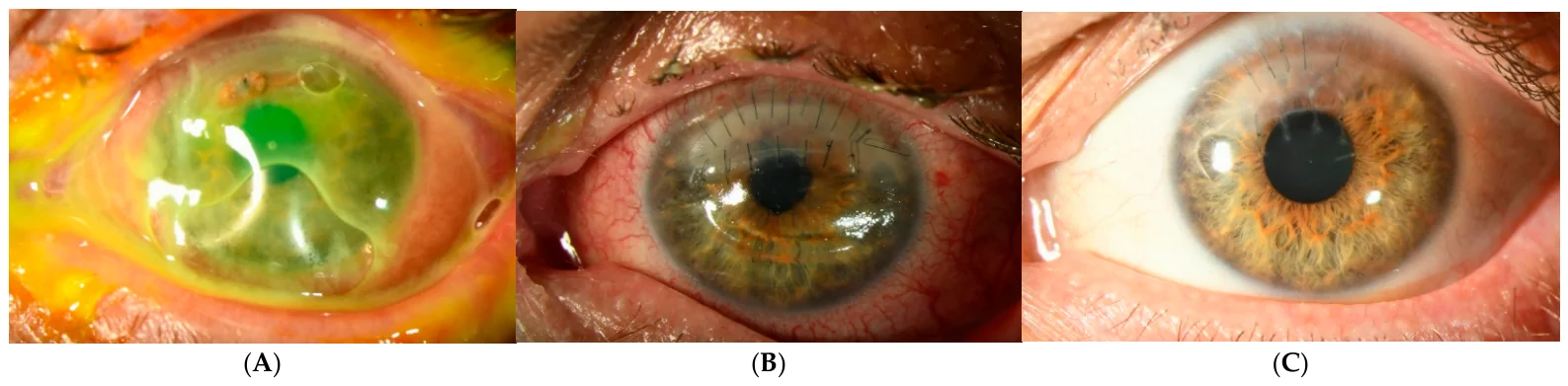
Case no. 14 who presented with corneal perforation due to gonococcal keratoconjunctivitis ((**A**): clinical presentation at initial visit, with inserted contact lens, (**B**): 3 days after customized DALK, (**C**): 10 months after customized DALK).

Surgical intervention was required in one case (customized deep anterior lamellar keratoplasty (DALK) for corneal perforation with iris prolapse, depicted in Figure 4). In case no. 1, a phototherapeutic keratectomy was performed during treatment to remove superficial corneal opacities and promote more regular epithelialization.

Complications occurred in seven (41.1%) patients and included symblepharon, conjunctival abscess, eyelid cellulitis, persistent epithelial defects, severe keratouveitis, and corneal perforation. All complications were managed conservatively except for the above case requiring surgical intervention. In one patient, gonococcal conjunctivitis was diagnosed in the setting of prior evisceration with an ocular prosthesis and was associated with marked eyelid cellulitis (case no. 10).

### 3.3. Chlamydial Conjunctivitis

**Figure 5:**
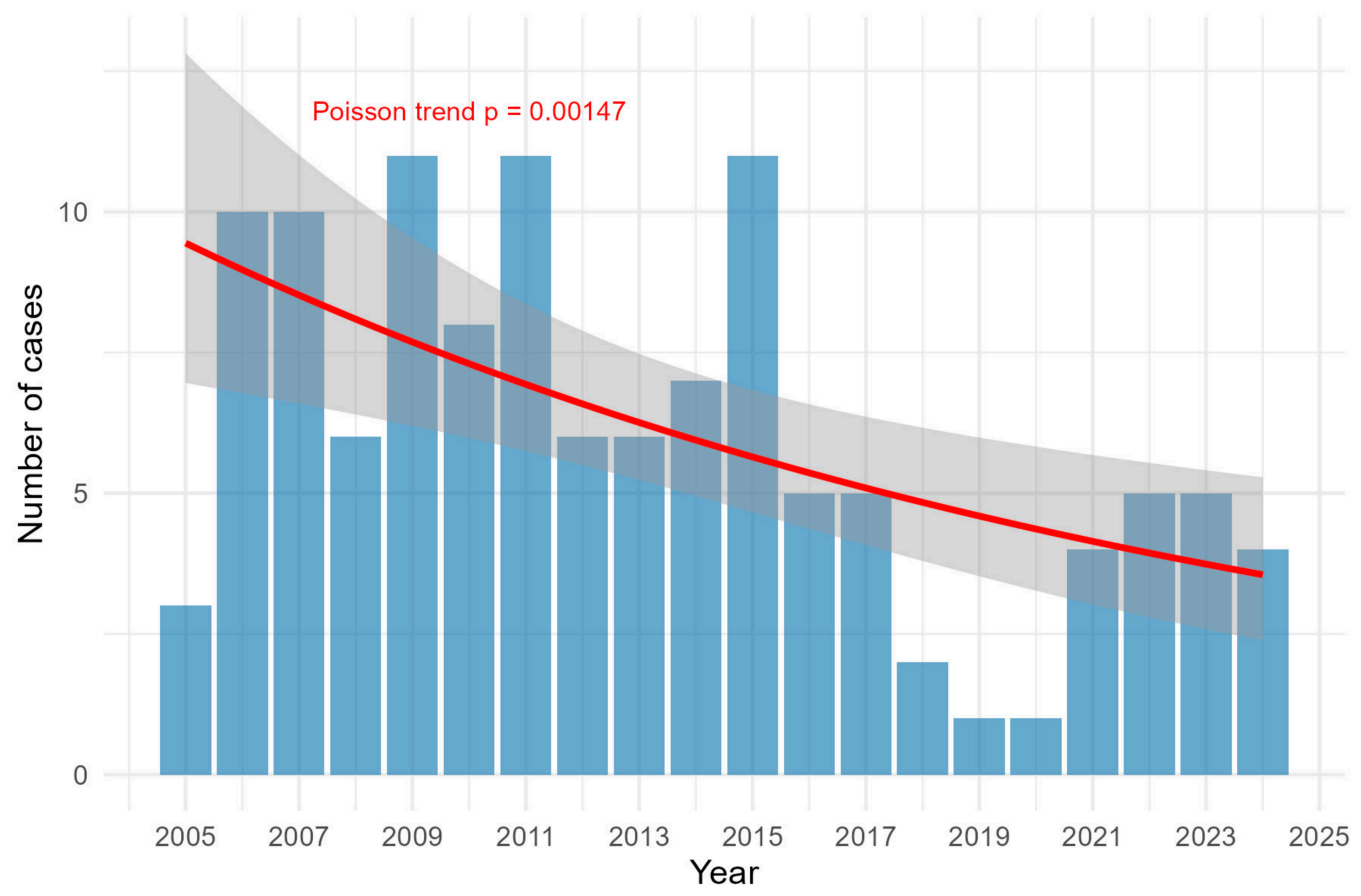
Annual number of chlamydial conjunctivitis cases (2005–2025) with fitted Poisson trend line (red).

A marked downward trend in chlamydial cases was observed over the study period from 2005 to 2025, displayed in Figure 5. Poisson regression estimated an average annual decrease of 5.0% (95% CI: 2.0% to 8.0%, *p* = 0.0015). The Mann–Kendall test confirmed a monotonic decline (τ = –0.433, *p* = 0.0106).

Change-point analysis (Pettitt test) identified a significant structural break in 2015 (*p* = 0.009). When the data were divided into two epochs, the mean annual case count fell from 8.1 cases/year (2005–2015) to 3.6 cases/year (2016–2025), a difference that was highly significant (Mann–Whitney U test, *p* = 0.00099) (Table 3).

### 3.4. Comparison Between Pathogens

**Figure 6:**
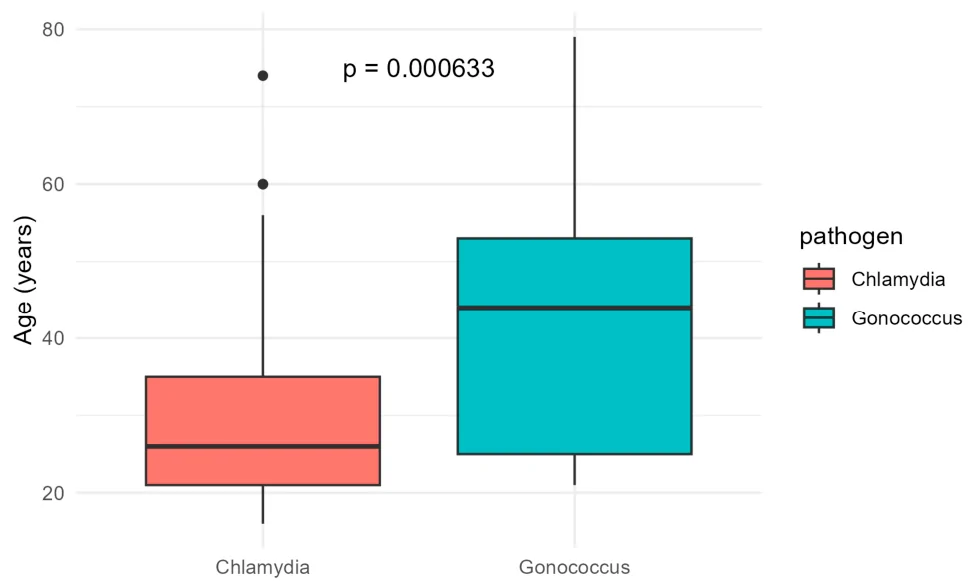
Boxplot comparing age distribution between chlamydia and gonococcus groups.

Gonococcus patients were significantly older than chlamydia patients (median 44 vs. 26 years, Mann–Whitney U = 501, *p* = 0.00063). Although the proportion of males was higher in the gonococcus group (88.2%) than in the chlamydia group (68.6%), the difference was not statistically significant (Fisher’s exact test, *p* = 0.151; odds ratio for male sex = 3.41, 95% CI: 0.73–32.2). The detailed results are depicted in Table 4 and Figure 6.

## 4. Discussion

Sexually transmitted bacterial (kerato-)conjunctivitis, particularly gonococcal and chlamydial infection, is an uncommon but clinically significant ocular disease requiring prompt recognition and appropriate antimicrobial treatment to prevent irreversible visual impairment, particularly in cases of gonococcal keratoconjunctivitis. In this retrospective study, we observed a marked and statistically significant increase in gonococcal eye infections over the study period, while chlamydial conjunctivitis showed a significant downward trend.

In accordance with previous reports [8,32], our findings show that gonococcal infections were predominantly associated with hyperacute purulent conjunctivitis and periorbital swelling. Notably, more than half of our patients already exhibited corneal involvement at presentation, underscoring the rapid progression and destructive potential of *N. gonorrhoeae* [9,10]. Severe complications, including corneal perforation, although rare [33,34,35], highlight the importance of early diagnosis and urgent ophthalmologic referral. The relatively high rate of correct initial clinical suspicion in our cohort suggests increasing awareness among referring physicians; however, delayed presentation remains a critical risk factor for adverse outcomes.

The demographic characteristics of our cohort largely align with the existing literature, particularly the marked male predominance [6,11,32,36]. However, the median age of our cohort was substantially higher than previously reported [6,11,32]. This discrepancy may reflect evolving sexual behavior patterns, differences in healthcare-seeking behavior, or referral bias inherent to a tertiary care center. The frequent reporting of concomitant urogenital symptoms and high-risk sexual behavior further emphasizes the systemic nature of the infection and supports the need for interdisciplinary management involving ophthalmology, infectious diseases, and gynecology or urology.

From a clinical perspective, our findings reinforce current treatment recommendations, particularly the central role of systemic ceftriaxone therapy. At the same time, the variability in adjunctive topical treatment observed in our cohort reflects the lack of standardized protocols and highlights the need for individualized management based on disease severity. The relatively high proportion of complicated disease courses and the clustering of severe cases in the most recent years further underscore the clinical relevance of the observed increase in gonococcal eye infections. Despite the potential severity of these infections, visual outcomes in our cohort were generally favorable, with normalization of visual acuity in over half of the cases.

The general global trend of rising incidence of gonorrhoea and chlamydia infections is also reflected in Switzerland. Since 1988, the Swiss Federal Office of Public Health (FOPH) has been collecting data on chlamydia and gonorrhoea cases through the mandatory notification system, recording information on sex, date of birth, and canton of residence. This allows for the publication of annual key statistics on the infection prevalence [37]. The 2024 epidemiological assessment once again confirmed the increase in reported gonorrhoea cases that has been observed for several years. In 2024, the incidence was 75.6 per 100,000 residents, representing an 11.6% increase compared with the previous year. Since 2001, the number of cases has increased 13.8-fold [29]. It is important to consider that our tertiary referral center serves the metropolitan region of Zurich, which consistently has the highest reported incidence of gonorrhoea in Switzerland [29].

Recent epidemiological data also indicate a rising incidence of gonococcal keratoconjunctivitis in Europe. A Spanish nationwide analysis reported an upward trend in adult gonococcal keratoconjunctivitis between 2017 and 2023, with a marked peak of incidence rate in 2023 [12]. Similarly, a descriptive, retrospective case series from two tertiary eye hospitals in Western Europe (Moorfields Eye Hospital, London and Rotterdam Eye Hospital, Netherlands) documented a substantial increase in adult gonococcal keratoconjunctivitis cases in 2023 (11 cases in 2023 vs. ≤3 cases per year in 2017–2022) [11]. Interestingly, our study also identified a peak in gonococcal eye infections in 2023. Although the underlying reasons remain uncertain, the temporal concordance with observations from other European centers supports the possibility that the increase represents a broader epidemiological phenomenon rather than an isolated local finding. These findings highlight the importance of continued surveillance and antimicrobial susceptibility monitoring.

The number of reported urogenital chlamydia cases in Switzerland increased steadily between 2000 and 2022; however, this trend was interrupted by a slight decline in 2023, followed by a stabilization of case numbers in 2024 [29]. The number of cases among women peaked in 2016 and has slightly decreased since 2023. In men, case numbers continue to increase, albeit at a slower rate since 2023 [29]. In contrast, we did not observe a corresponding increase in chlamydial conjunctivitis during the study period; rather, the number of cases diagnosed at our tertiary referral center declined. The underlying cause of this discrepancy remains unclear and is likely multifactorial. Neither our diagnostic approach, which remained restricted to symptomatic patients, nor the referral pattern to our institution changed substantially during the study period. One speculative explanation is increasing awareness of urogenital chlamydial infection among primary care physicians and other first-line healthcare providers, leading to earlier diagnosis and treatment before ocular manifestations develop or require referral to a tertiary ophthalmic center. However, our study did not collect data on changes in primary-care awareness, time to diagnosis, or treatment timing, and this hypothesis therefore cannot be directly substantiated by our data. In addition, the increase in reported urogenital chlamydia cases may partly reflect intensified screening rather than a true increase in disease incidence [38]. Despite the lack of data on the total number of tests performed, the FOPH also assumes that the long-term upward trend is attributable to increased testing [29]. Because urogenital chlamydial infection is frequently asymptomatic [39,40], expanded testing is likely to identify a larger number of previously undetected infections, creating a stronger surveillance effect than for gonorrhoea, which is more often symptomatic [41]. It is likewise possible, but not demonstrable from our data, that earlier identification and treatment of these asymptomatic infections may reduce transmission and subsequent autoinoculation to the eye. These explanations should therefore be regarded as hypotheses rather than established causes of the observed decline in chlamydial conjunctivitis.

Although gonococcal and chlamydial infections are notifiable diseases in Switzerland, current laboratory and physician reporting forms do not record ocular manifestations or involvement. The absence of corresponding epidemiological data has been confirmed by the FOPH. Our study therefore provides an important contribution by addressing a relevant gap in the existing surveillance data regarding ocular involvement and clinical presentation of gonococcal and chlamydial infections.

This study has several limitations. The observed rise in gonococcal cases may partly reflect increased testing volume or heightened clinical and referral awareness rather than a true increase in disease burden. Although two diagnostic workflows were used during the study period (Cobas 4800 and, additionally, the QIAsymphony/LightCycler workflow), the latter was established approximately 10 years ago and used in parallel with the Cobas 4800 rather than replacing it. The two workflows have comparable analytical sensitivity for the detection of *C. trachomatis* and *N. gonorrhoeae*. Therefore, a systematic change in diagnostic methodology is unlikely to explain the observed increase, although differences related to the use of the two workflows cannot be completely excluded. We did not have complete historical data on the total number of conjunctival swabs performed throughout the study period or comparable data on genitourinary gonorrhea notifications within the same catchment area. The available data of our department on conjunctival testing are provided in Appendix A Table A3. Although increased testing may have contributed to increased case ascertainment, this explanation alone may not fully account for the observed rise, as ocular gonococcal infection is generally not self-limiting and may rapidly progress if untreated. Nevertheless, previously unrecognized ocular infections may have been incidentally treated in patients receiving treatment for concomitant urogenital gonorrhea. These possibilities cannot be distinguished retrospectively, and ascertainment bias therefore remains an important limitation of the study.

Similarly, the reasons for the observed decline in chlamydial conjunctivitis remain uncertain. Although increased primary-care awareness, earlier diagnosis, and treatment of urogenital chlamydial infections may have contributed, these explanations are speculative and were not directly assessed in our study. We had no data on changes in primary-care awareness, screening practices, time to diagnosis, or time to treatment that would allow these hypotheses to be evaluated.

The retrospective design of the study introduces the risk of incomplete data and reporting bias. Furthermore, the single-center setting and the relatively small number of gonococcal cases limit the generalizability of our findings. Sexual history was not systematically documented, precluding robust analysis of risk factors, and follow-up data were not available for all patients. Therefore, causal inferences and detailed outcome assessments remain limited.

Future research should focus on prospective, multicenter studies to better characterize epidemiological trends and risk factors, as well as to evaluate standardized diagnostic and therapeutic approaches. In addition, ongoing surveillance and antimicrobial resistance monitoring are essential, considering the increasing global burden of gonococcal infections.

## 5. Conclusions

In conclusion, our study demonstrates a significant recent increase in gonococcal eye infections, in contrast to decreasing rates of chlamydial conjunctivitis. Given the potentially rapid and severe course of gonococcal keratoconjunctivitis, heightened clinical awareness, early diagnosis, and prompt systemic treatment are essential to preserving visual function.

## Figures and Tables

**Table 1:** Descriptive statistics.

Pathogen	*n*	Female (*n*)	Female (%)	Male (*n*)	Male (%)	Age Median	Age IQR	Age Range
Chlamydia	121	38	31.4	83	68.6	26	20–34	16–74
Gonococcus	17	2	11.8	15	88.2	44	30–58	21–79

**Table 2:** Gonococcal conjunctivitis/keratoconjunctivitis: period comparison.

Period	Years	Cases	Annual Rate
2005–2020	16	5	0.31
2021–2025	5	12	2.4
IRR (2021–2025 vs. 2005–2020)		7.68 (95% CI: 2.52–27.83)	*p* = 0.00006

Clinical characteristics of the 17 individual cases are listed in the Table A2. Percentages in the text are based on the total number of patients (*n* = 17).

**Table 3:** Chlamydial Conjunctivitis: Trend Analysis.

Test	Result
Poisson regression (annual % change)	−5% (95% CI: −8 to −2%), *p* = 0.0015
Mann–Kendall τ	τ = −0.433, *p* = 0.010578
Pettitt change-point (year)	2015, *p* = 0.009
Epoch comparison: 2005–2015 mean	8.1 cases/year
Epoch comparison: 2016–2025 mean	3.6 cases/year

**Table 4:** Comparison between the chlamydia and gonococcus cohorts.

Variable	Chlamydia	Gonococcus	*p*_Value
Age (median, IQR)	26 (14)	44 (28)	
Age *p*-value			0.00063262
Gender (% male)	68.6%	88.2%	
Gender *p*-value			0.15134
Odds ratio (male vs. female)			3.41 (95% CI: 0.73–32.21)

## Data Availability

Data will be made available upon request to the corresponding author (due to ethical reasons).

## Abbreviations

The following abbreviations are used in this manuscript:

CI	Confidence interval
CT	Computed Tomography
*C. trachomatis*	*Chlamydia trachomatis*
DALK	Deep anterior lamellar keratoplasty
FOPH	Federal Office of Public Health
IRR	Incidence rate ratio
IQR	Interquartile range
MSM	Men Who Have Sex with Men
*N. gonorrhoeae*	*Neisseria gonorrhoeae*
No.	Number
PCR	Polymerase Chain Reaction
STD	Sexually Transmitted Disease
WHO	World Health Organization

## Appendix A

**Table A1:** Global recommendations for the treatment of gonococcal conjunctivitis in adults/adolescents (CDC—Centers for Disease Control and Prevention, ECDS—European Center for Disease Prevention and Control, BASHH—British Association for Sexual Health and HIV, IM—intramuscular, IV—intravenous, PO—per os).

Current Guideline	Preferred Treatment
United States (CDC, 2021) [42]	Ceftriaxone 1 g IM single dose (+ consider one-time lavage of the infected eye with saline solution)
European (ECDC, 2025) [43]	Ceftriaxone (1 g) plus Azithromycin (2 g) dual therapy or Ceftriaxone monotherapy (1 g) (not specified whether IM, IV or PO)
Germany [44]	Ceftriaxone 2 g IM or IV daily for 3 days plus Azithromycin 1.5 g PO single dose
United Kingdom (BASHH, 2025) [5]	Ceftriaxone 1 g IM single dose (+ adjunctive cefuroxime 5% eyedrops, additional lavage of the infected eye with saline solution if copious discharge is present)

**Table A2:** Clinical characteristics of patients with gonococcal eye infections 2005–2025.

No	Eye	Year	Age	Sex	Typical Sexual History	PCR	Culture	Urogenital Symptoms	Proven Urogenital Co-Infection	VA on Presentation (Snellen Decimal)	Corneal Involvement	CT	Treatment Delay, Days	Systemic Treatment	Topical Treatment	Complications	VA on Last Follow Up
1	OU	2005	47	M	no	pos	pos	no	no	OD correct light perception, OS s.c. FC at 30 cm	yes (OU corneal infiltrate and OD stromal defect)	no	0	Ceftriaxon 2 g IV daily (14 days)	Ofloxacin 0.3%, Ceftazidim 5%, Dexamethason 0.1%, autologous eye serum drops, heparin, contact lense	yes (corneal stromal defect)	OD c.c. 0.2 OS c.c. 1.0
2	OS	2008	23	M	NA	NA	pos	NA	no	NA	no	yes	0	Co-Amoxicillin 2.2 g IV (2 days), Co-Amoxicillin 2 × 1 g PO (7 days)	Ofloxacin 0.3%	no	s.c. 1.0
3	OD	2012	25	M	yes (MSM)	pos	pos	no	no	NA (due to swelling, 2nd day HM at 0.5 m s.c.)	yes (corneal erosion)	no	1	Ceftriaxon 1 g IV (two doses), Azithromycin 1 g PO (single dose)	Ofloxacin 0.3%, Dexamethason 0.1%, Prednisolon pivalate 0.5%, Tobramycin/Dexamethason 0.3%/0.1%	yes (symblepharon inferior)	c.c. 0.5
4	OS	2013	79	M	no	NA	pos	no	no	s.c. HM at 0.5 m	yes (corneal ulcer)	no	12	Ceftriaxon 1 g IV daily (7 days), Azithromycin 1 g PO (single dose), Vibramycin PO (5 weeks)	Ofloxacin/Ceftazidim (0.15%/2.5%), Dexamethason 0.1%, autologous eye serum drops	yes (concomitant uveitis, persistent corneal erosion)	c.c. FC 0.2 m
5	OS	2013	25	F	yes	pos	neg	yes	NA	s.c. 0.5 (PH 0.6)	yes (corneal infiltrates)	no	11	Ceftriaxon 1 g IM (single dose), Azithromycin 1 g PO single dose	Ofloxacin 0.3%, Dexamethason 0.1%	no	s.c. 0.9
6	OU	2021	63	M	no	pos	pos	no	no	OD c.c. FC at 0.2 m OS c.c. 1.0	no	no	1	Ceftriaxon 1 g IV (two doses)	Ofloxacin/Ceftazidim (0.15%/2.5%), Ofloxacin 0.3%,	no	OU c.c. 1.0
7	OU	2022	65	M	yes (MSM)	pos	pos	no	no	OD s.c. 0.3 (PH 0.6) OS NA (due to swelling)	no	yes	0	Ceftriaxon 1 g IM (single dose)	Ofloxacin/Ceftazidim (0.15%/2.5%), Ofloxacin 0.3%	yes (Conjunctival abscess)	OD s.c. 0.8 OS s.c. 0.6
8	OD	2023	25	M	NA	NA	pos	NA	NA	NA	NA	NA	NA	NA	NA	NA	NA
9	OD	2023	53	M	yes	pos	NA	yes	yes	c.c. 0.6	yes (corneal erosion)	no	1	Ceftriaxon 1 g IM (single dose), Vibramycin 100 mg 2 × daily PO (1 week)	Moxifloxacin 5 mg/mL, Ofloxacin 0.3%, Dexamethason 0.1%	no	c.c. 1.0
10	OD	2023	44	M	no	pos	NA	no	no	NA	NA	yes	6	Co-Amoxicillin PO initially, Ceftriaxon 1 g IM (single dose)	Moxifloxacin 0.5%	yes (pre-septal cellulitis status post evisceration with ocular prosthesis)	NA
11	OS	2023	74	M	NA	pos	neg	yes	no	s.c. 0.3	no	no	1	Ceftriaxon 1 g IM (single dose), Vibramycin 2 × 100 mg PO daily (1 week)	Moxifloxacin 0.5%	no	s.c. 0.8
12	OD	2023	21	F	yes	pos	pos	no	no	s.c. 1.0	o	no	3	Ceftriaxon 1 g IM (single dose)	Moxifloxacin 0.5%	no	s.c. 1.0
13	OS	2023	51	M	yes (MSM)	pos	neg	no	yes	c.c. 0.8	yes (corneal infiltrates)	no	1	Ceftriaxon 1 g IM (single dose), Vibramycin 2 × 100 mg PO daily (1 week)	Ofloxacin/Ceftazidim (0.15%/2.5%), Ofloxacin 0.3%,	no	c.c. 0.8
14	OS	2024	47	M	no	pos	neg	yes	yes	s.c. 0.25	yes (corneal perforation)	no	0	Meropenem 1 g IV (2 doses), Doxycyclin 1 × 100 mg IV, Aciclovir 1 × 400 mg IV, Ceftriaxon 2 g IV (1 x daily for 10 days), then 1 × 1 g IM	Ofloxacin/Ceftazidim (0.15%/2.5%)	yes (corneal perforation)	s.c. 0.4
15	OD	2025	38	M	no	pos	pos	no	no	s.c. 0.16 (PH 0.3)	yes (corneal clouding)	yes	2	Initially Clindamycin 4 × 300 mg (1 day), then Ceftriaxon 2 g IV/24 h (7 days), Metronidazol 3 × 500 mg PO (7 days), Azithromyzin 1 g PO (single dose)	Ofloxacin/Ceftazidim (0.15%/2.5%), Ofloxacin 0.3%	yes (persistent corneal erosion)	s.c. 0.5
16	OS	2025	43	M	no	pos	neg	no	yes	s.c. 1.0	no	yes	3	Ceftriaxon 2 g IV (3 doses), then 1 g IM (single dose)	Moxifloxacin 0.5%	no	s.c. 1.0
17	OD	2025	36	M	yes (MSM)	pos	neg	no	no	s.c. 0.25	yes (corneal stromal and epithelial edema)	yes	1	Co-Amoxicillin 2.2 g IV (single dose), Ceftriaxon 2 g IV (7 days), Metronidazol 3 × 500 mg daily PO (7 days)	Ofloxacin/Ceftazidim (0.15%/2.5%)	no	s.c. 1.0

Abbreviations: VA—visual acuity, CT—computed tomography, M—male, F—female, MSM—men who have sex with men, NA—not available, OD—oculus dexter, OS—oculus sinister, HM—hand movements, m—meters, FC—finger counting, s.c.—sine correctione, c.c.—cum correctione, IM—intramuscular, IV—intravenous, PO—per os, pos—positive, neg—negative, PH—pinhole.

**Table A3:** Total number of conjunctival swabs for *N. gonorrhoeae* performed per year.

Year	Total Number of Conjunctival Swabs
2005	30
2006	24
2007	9
2008	9
2009	7
2010	21
2011	44
2012	76
2013	36
2014	52
2015	35
2016	25
2017	35
2018	25
2019	25
2020	11
2021	19
2022	54
2023	51
2024	69
2025	55
